# Potent antagonistic activity of Egyptian *Lactobacillus plantarum* against multiresistant and virulent food-associated pathogens

**DOI:** 10.3389/fmicb.2015.00347

**Published:** 2015-05-12

**Authors:** Lamiaa A. Al-Madboly, Abeer K. Abdullah

**Affiliations:** ^1^Department of Pharmaceutical Microbiology, Faculty of Pharmacy, Tanta UniversityTanta, Egypt; ^2^Department of Microbiology and Immunology, Faculty of Pharmacy, Al-Azhar UniversityCairo, Egypt

**Keywords:** *Lactobacillus plantarum*, fermented milk, antibacterial activity, virulent, multiresistant, stressors

## Abstract

Recent years have shown a growing interest to replace the administration of antibiotics with the application of probiotics. The aim of our investigation was to screen for promising strains with broad antimicrobial activity and also more resistant to the challenges met in the gastrointestinal tract. In our study, only 32 out of 50 (64%) probiotic isolates showed antagonistic activity against certain major extensively and pandrug-resistant Gram-positive and -negative food-borne pathogens. Fifteen *L. plantarum* isolates had a broad antibacterial spectrum. Among these isolates, only five presented potent antibacterial activity relative to previous studies. The recorded inhibition zone diameter ranged from 25 to 44 mm. Pronounced cell-free supernatant activities (6400–25,600 AU/ml) were commonly detected at the end of the logarithmic phase at 37°C. A marked increase in the range of activity (12,800–51,200 AU/ml) was recorded after the addition of 0.9% Na Cl to the media. Moreover, subjecting these isolates to different stressors, including high temperature, low pH, and different concentrations of bile and Na Cl, revealed different responses, and only two out of the five *L. plantarum* isolates showed marked resistance to all of the stress factors. Accordingly, this study highlights the intense and broad antagonistic activity induced by *L. plantarum* against various food associated pathogens, and their ability to resist different stressors suggests that they can be used in the food and pharmaceutical industry.

## Introduction

Most foods are nonsterile and can be contaminated with saprophytic and pathogenic microorganisms. Saprophytic microorganisms utilize substrates in food as well as the metabolites they produce causing microbial spoilage. Pathogens that contaminate food cause food-borne illness such as gastritis, enteritis, peptic ulcer and ulcerative colitis, when they enter the gastrointestinal tract. To ensure microbial food safety to public health, there is currently a trend to enrich food with lactic acid bacteria (LAB) with high antimicrobial activity against pathogenic and saprophytic microorganisms (Samot and Badet, 2013).

LAB are indigenous inhabitants of the human intestinal tract. They have a long history of traditional use in many industrial and artisanal plant, meat, and dairy fermentation. Some LAB strains have clearly been shown to exert beneficial health effects so that they are commonly marketed as probiotics (Hamon et al., 2011). They play a significant physiological role in the maintenance of the ecological balance due to the production of lactic acid which is responsible for the low pH level observed in the gastrointestinal tract. In addition, these bacteria also produce other inhibitory substances, such as hydrogen peroxide, bacteriocins and some organic acids. Other mechanisms proposed for their microbial antagonism are competition for nutrition, inhibition of the adhesion of pathogens to surfaces and stimulation of the immune system (Voravuthikunchai et al., 2006). Additionally, they may prevent cancer, reduce the risk of inflammatory bowel movements, improve the digestion of proteins and fats, synthesize vitamins, detox and protect from toxins. Moreover, LAB beneficial health effects are known to be strain specific (Hobbs, 2000; Hamon et al., 2011).

Lactobacilli, despite their origin, may potentially inhibit the growth of pathogens, including *Escherichia coli, Staphylococcus aureus*, and *Candida albicans* (Verdenelli et al., 2009). Previous studies have revealed that *Lactobacillus reuteri, L. fermentum*, and *L. plantarum* isolated from a healthy vaginal ecosystem, significantly inhibit methicillin-resistant *S. aureus*. It has also been demonstrated that *L. rhamnosus* has the capacity to displace and kill *S. aureus* adhering to the human intestinal mucus by 39–44% (Vesterlund et al., 2006; Voravuthikunchai et al., 2006; Nawaz et al., 2008; Maxton et al., 2013).

This study searched for additional promising probiotic strains with strong antibacterial activity against some common food-associated pathogens. Additionally, our work aimed to assess the degree of tolerance of the identified promising strains to different stress factors that could be faced during passage in the gastrointestinal tract or industrial fermentors.

## Materials and methods

### Isolation and identification of lactic acid bacteria

A total of 64 homemade fermented milk “yogurt” samples were collected from different rural areas in the Delta region in Egypt over a period of 6 months (from September, 2013 until February, 2014). Approximately 10 g of each sample was separately suspended in 50 ml of 0.9% w/v normal saline and then vigorously mixed on vortex (Julabo, Germany). Serial dilutions of the samples were prepared and then swab-inoculated on de Man Rogosa Sharpe (MRS) agar plates (DIFCO, USA). The plates were incubated anaerobically in anaerobic jars using GasPak (Oxoid, USA) at 37°C for 72 h to obtain colonies (De Man et al., 1960).

LAB were identified based on their colony morphology, Gram-staining, oxidase and milk coagulation activities, and motility and catalase test results. Identification at the species level was performed for 15 lactobacillus isolates using API-50 CHL (Biomueirex, France). The results were recorded as fermentation profiles and interpreted using the Biomueirex database (France). For more confirmation, the most potent five *L. plantarum* isolates were further identified using the 16S rRNA sequencing. After extracting the total genomic DNA from the isolates using GeneJET™ DNA extraction kit (Thermo Scientific, USA), it was subjected to amplification using PCR technique. The forward primer used was 5′-AGA GTT TGA TCC TGG CTC AG-3′ and the reverse one was 5′-GGT TAC CTT GTT ACG ACT T-3′. The PCR amplifications were performed in a TC-3000G Thermocycler (TECHNE, UK) using the following PCR program: 10 min at 95°C; 30 cycles of 30 s at 95°C, 60 s at 65°C, 90 s at 72°C and 10 min at 72°C. The PCR products were run on a 0.9% A 9539 agarose gel (Sigma, USA). The amplicons were then purified with the GeneJET™ PCR purification kit (Thermo Scientific, USA). Determination of the base sequences was carried out using ABI 3730xl DNA sequencer. The generated sequences were subjected to similarity screening in the database using the BLASTN; Basic local alignment and search tool of nucleotides, website: http://blast.ncbi.nlm.nih.gov/Blast.cgi. The isolates were then identified based on the results of the analysis. Accession no. (NR 115605.1) and (NR 075041.1).

### Test pathogens

The antimicrobial activity of LAB was studied against eight virulent indicator strains, including enterotoxigenic *S. aureus*, gelatinase producing *Enterococcus faecalis* (OG1RF), enteroaggregative *E. coli*, enteroinvasive *Shigella flexneri*, and *Salmonella enterica* subsp. *enterica serovar Typhi* (ATCC 6539), which were obtained from the Department of Microbiology at the Faculty of Pharmacy of Tanta University, Egypt. *Lactobacillus casei* (ATCC 20011) was obtained from the Egyptian Microbial Culture Collection at Cairo Microbiological Resources Center (MIRCEN), Faculty of Agriculture, Ain Shams University, Egypt.

The indicator strains were tested for their sensitivity toward different antimicrobial agents using the disk diffusion method (Bauer et al., 1966). The inhibition zone appeared around the antibiotic discs (Oxoid, USA) was measured in “mm,” and the results were interpreted based on CLSI standards (CLSI, 2014).

### Assessment of antibacterial activity of lactic acid bacteria against food-borne pathogens

The antibacterial spectrum of the supernatant from lactobacilli and streptococci was determined using well diffusion method. The cell-free supernatant (CFS) from a 48-h culture of probiotics in MRS broth (Oxoid, UK) was filter-sterilized by passage through a 0.45-μm-pore-size Whatman membrane filter (Sigma, USA). Aliquots of the sterile supernatant were placed in 5-mm-diameter wells that had been cut in Mueller-Hinton agar plates previously seeded with the indicator strains. Wells without CFS were used as negative controls, and *L. casei* CSF was used as a positive control. After 12–18 h of incubation, the diameters of the inhibition zones were measured in (mm). Each measured diameter represents the results of three separate experiments (Ivanova et al., 2000).

### Growth curves of *L. plantarum* isolates and CFS activity

It was important to ascertain the growth time of the LAB to determine the stopping point for fermentation and to provide the time at which bacteriocin production and activity, if present, were maximal. After inoculation of activated LAB into MRS broth, the culture was incubated at 37°C in a shaking incubator, and aliquots were taken at 0, 2, 4, 6, 8, 10, 18, 24, 40, 44, 48, and 72 h. The optical density was measured at 660 nm. At the end of each incubation period, the CFS activity was observed by inoculating the culture supernatant against an indicator organism. The antimicrobial activity of the CFS is expressed in arbitrary units (AU/ml), and 1 AU was defined as the reciprocal of the highest level of dilution resulting in a clear zone of growth inhibition multiplied by 100 (Todorov and Dicks, 2005a).

The effect of different concentrations of the CFS of the *L. plantarum* (L_1_) isolate on the growth of indicator strains was also studied. After 4 h of incubation, the CFS was added to culture broth containing the indicator strain. At appropriate intervals, the OD_620_ was recorded, and aliquots were cultured on nutrient agar plates to assess the bacteriostatic or bactericidal activity of the CFS (Todorov, 2008).

### Effect of different stress factors on bacterial growth

According to the results of antibacterial activity, the five *L. plantarum* isolates that showed the highest inhibition zone diameters were thus selected and subjected to different stress factors.

#### Higher temperature and hyperosmolarity

According to Ivanova et al. (2000) and Todorov and Dicks (2005b), the biomass and antibacterial activity of the CFS were assessed at various temperatures (20, 30, 37, 50, 60, and 70°C), and in the presence of various concentrations of sodium chloride (0.1, 0.9, 2, 4, 6, and 7.5%).

#### Acid tolerance test

The method used to evaluate the viability of the cells under acidic stress in this study was adapted from Conway et al. (1987) and Tsai et al. (2007). Various simulated GI conditions were achieved by subjecting the samples to different pH levels for a designated incubation time. Huang and Adams (2004) described the use of sterile PBS adjusted to different pH values for the study of the acid tolerance of the microorganisms. Hence, the pH of PBS was adjusted to pH 1.5, 3.0, and 7.2 (control) using 1 M HCl. In addition, the test microorganism was subjected to three incubation periods of 0, 1.5, and 3.0 h. Initially (at 0 h), 1.0 ml of the sample was inoculated into the universal bottle containing 9 ml of PBS at pH 1.5, then mixed thoroughly and serially diluted with sterilized PBS pH 7.2 in microcentrifuge tubes. The appropriate dilution factor was determined, and the serial dilutions of the culture were plated on MRS agar then incubated anaerobically at 37°C for 48 h. Each assay was performed in triplicate. The same procedure was repeated for pH 3.0 and pH 7.2 using the same experimental conditions for the 0-h sample. The aforementioned process was performed for three incubation periods (0, 1.5, and 3.0 h). The acid tolerance was estimated by comparing the viable cell counts in all of the MRS agar plates after 48 h of incubation.

#### Bile tolerance test

The effects of bile on the growth of probiotic strains were determined according to the procedure described by Tsai et al. (2007). A series of bile concentrations were employed in this study based on the fluctuation in the bile concentration over time. Broth with 0% bile served as a control in the study. The bile tolerance test was commenced after 3 h of acid pretreatment, at which point 5 ml of each sample was pipetted from the universal bottles incubated at pH 1.5, 3.0, or 7.2 into three pH-labeled centrifuge tubes. The samples were centrifuged at 4000 rpm for 10 min. The supernatants were discarded, and the pellets were washed with PBS at pH 7.2. The samples were centrifuged again, and the supernatants were once again discarded. The three remaining concentrates were then re-suspended in MRS broth. The next procedure involved the inoculation of 1 ml of the suspensions at pH 1.5 into 9 ml of MRS broth with different bile concentrations (0, 0.5, and 2.0%). This step was repeated for the mixtures incubated at pH 3.0 and 7.2. The cell cultures in MRS broth were incubated anaerobically at 37°C for 24 h. Subsequently, 0.1 ml was pipetted out from each culture, and serial dilutions were performed for plating (in triplicate). All of the plates were incubated anaerobically at 37°C for 48 h. The bile tolerance was determined by comparing the viable cell counts on MRS agar with and without bile salt. All of the samples were analyzed in triplicate, and all of the experiments were repeated three times. The data obtained from the study were expressed in terms of log_10_ CFU/ml and analyzed as the means ± standard deviation (SD).

### Statistical analysis

The values were expressed as the means (from three replicates) ± standard deviation (SD). The differences between groups were determined by one-way analysis of variance (ANOVA) considering a value of α < 0.05. We accepted the null hypothesis (H_0_) according to Levene's test. Because P < α, a significant difference was found between the means of the groups. In addition, equal variances were assumed according to the *post-hoc* test. The data were analyzed using SPSS (version 17).

## Results and discussion

Probiotic microorganisms are extensively used in a wide range of applications such as prevention of food poisoning or treatment of certain gastrointestinal disorders because most health effects attributed to them are related directly or indirectly to the gastrointestinal tract, i.e., mediated by the immune system (Coconnier et al., 1997; Marteau et al., 2001; Zvanych et al., 2014). In this study, a total of 50 (78.1%) isolates of LAB were recovered from 64 fermented milk samples collected from different rural areas in the Delta region. All of these isolates were subjected to Gram staining, which revealed Gram-positive bacteria, including lactobacilli and streptococci. All of them were oxidase-negative, nonmotile, and catalase-negative and could coagulate milk. The colony morphology was also considered. The lactobacilli presented creamy-white color, circular, convex and moist colonies with smooth edges, whereas the pinpoint colonies observed were related to streptococci. These preliminary tests made it possible to classify the isolates at the genus level into lactobacilli and streptococci. Yeast isolates were omitted from this study.

Good probiotics should present their antimicrobial actions particularly to the pathogens in the GI system (Samot and Badet, 2013). Accordingly, enterotoxigenic *S. aureus*, gelatinase-producing *E. faecalis*, enteroaggregative *E. coli*, enteroinvasive *Sh. flexneri* and *S. typhi* were used as indicator bacteria in our study because they are occasionally found as food-borne microorganisms that may cause gastroenteritis. Moreover, these isolates showed extensively and pandrug resistance patterns (XDR and PDR) according to Magiorakos et al. (2012) as presented in Table 1. The results of agar well diffusion test performed in this study showed that only 32 out of the 50 (64%) isolates presented a wide range of inhibitory effects against different multiple-drug-resistant food-borne pathogens. In addition, the CFS of 15 lactobacillus isolates denoted L_1_ through L_15_, showed high antimicrobial activity recorded as inhibition zone diameters in Table 2. The later isolates were identified at the species level as *L. plantarum* using API-50 CHL (Biomueirex, France) as well as the 16S rRNA sequencing. Moreover, we recorded that five out of the 15 (33.3%) *L. plantarum* isolates showed the most potent antibacterial activity against all test pathogens (Table 2).

**Table 1:** **Resistance patterns of the indicator strains**.

**Indicator strains**	**Resistance patterns^a^**
*S. aureus*	AM-AmC-RAD-Va-C-E-AZM-Th-SXT
*E. faecalis*	RAD-CTX-FEP-Va-NOR-E-Cf-LEV-C-ERY-AZM-Th
*Sh. Flexneri*	AM-AmC-RAD-CFX-CAZ-S-G-AK-NOR-Cf-C-E-T-TMP-Th-SXT
*E. coli*	AM-AmC-IPM-RAD-CFX-CAZ-S-G-AK-NOR-Cf-LEV-C-E-T-TMP-Th-SXT
*S. typhi*	AM-AmC-RAD-NOR-C-E-T-TMP-Th-SXT

a AM, ampicilli; AmC, ampicillin/clavulanic acid; IPM, imipenem; RAD, cephradine; CTX, cefotaxime; FEP, cefepime; Va, vancomycin; C, chloramphenicol; E, erythromycin; AZM, azithromycin; Th, sulfamethizole; SXT, Trimethoprime/Sulfamethoxazole; NOR, norfloxacin; Cf, ciprofloxacin; LEV, levofloxacin; S, streptomycin; G, gentamicin; K, amikacin; T, tetracycline; TMP, trimethoprime.

**Table 2:** **Evaluation of the growth inhibition of Gram-positive and Gram-negative microorganisms by *L. plantarum* isolates using the well direct diffusion assay**.

**Code of *L. plantarum* isolates**	**Inhibition zone diameters against indicator strains (mm)^a^**					**pH of supernatant^b^**
	***S. aureus***	***E. faecalis***	***E. coli***	***Sh. flexneri***	***S. typhi***	
L_1_	44 ± 0.03	39 ± 1.7	35 ± 0.07	30 ± 1.1	28 ± 0.66	4.5
L_2_	37 ± 0.4	34 ± 0.54	33 ± 0.55	29 ± 1.09	27 ± 0.76	4.5
L_3_	34 ± 0.96	35 ± 0.45	33 ± 0.34	28 ± 0.55	25 ± 0.76	4.6
L_4_	40 ± 0.5	38 ± 0.22	37 ± 0.43	30 ± 0.06	28 ± 0.66	4.5
L_5_	36 ± 0.66	32 ± 1.65	34 ± 1.02	29 ± 0.75	26 ± 0.98	4.3
L_6_	30 ± 0.45	30 ± 0.098	30 ± 1.02	26 ± 0.43	25 ± 0.65	4.5
L_7_	30 ± 0.55	30 ± 0.044	32 ± 2.33	26 ± 0.43	22 ± 0.43	4.2
L_8_	26 ± 1.06	30 ± 0.43	30 ± 0.98	26 ± 0.34	24 ± 0.76	4.5
L_9_	26 ± 0.89	29 ± 0.23	31 ± 0.98	19 ± 0.75	20 ± 0.75	4.6
L_10_	32 ± 0.76	27 ± 0.15	32 ± 0.77	26 ± 1.98	23 ± 0.86	4.2
L_11_	30 ± 0.32	21 ± 0.033	25 ± 1.23	16 ± 0.54	22 ± 1.76	4.2
L_12_	18 ± 0.33	20 ± 0.78	16 ± 0.9	17 ± 0.64	15 ± 1.44	4.3
L_13_	19 ± 0.09	24 ± 1.87	16 ± 0.8	15 ± 1.07	14 ± 0.54	4.3
L_14_	29 ± 0.061	24 ± 1.43	24 ± 0.63	14 ± 0.043	22 ± 0.54	4.5
L_15_	29 ± 0.33	22 ± 0.99	27 ± 0.04	15 ± 0.03	21 ± 0.88	4.3
*L. casei*	19 ± 0.011	17 ± 0.12	15 ± 0.32	12 ± 0.012	11 ± 0.011	4.5
ATCC 20011						

a Mean of the diameter and standard deviation. Data are representative of three trials.

b pH of lactobacillus supernatant after 24 h of incubation (pH of MRS broth before culture was 6.5 ± 2).

**Figure 1:**
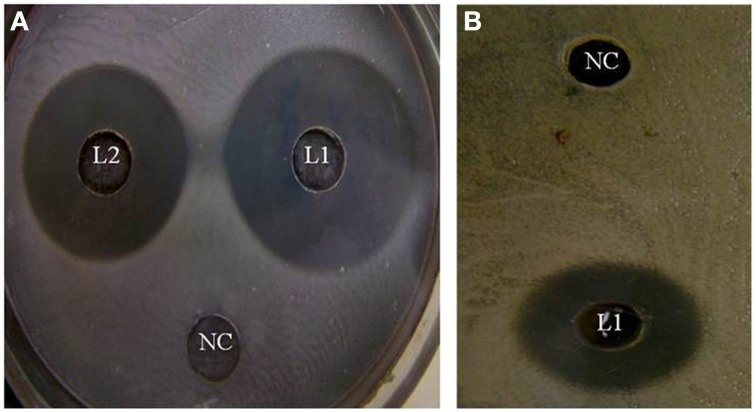
**Effect of the cell-free supernatants of different *L. plantarum isolates* on (A) enterotoxigenic *S. aureus* and (B) enteroaggregative *E. coli* showing different inhibition zones diameters.** NC, refers to the negative control.

In the study conducted by Maxton et al. (2013), maximum inhibition was observed by an *L. plantarum* strain against methicillin-resistant *S. aureus* pathogens, and the highest inhibition zone recorded was 15 mm. It is worth mentioning that the *L. plantarum* isolated and tested in the present study showed higher inhibition zone diameters ranging from 36 to 44 mm against a vancomycin-resistant virulent *S. aureus* isolate. The maximum value was recorded for L_1_ strain, which presented an approximately three-fold increase in the diameter indicating that this isolate was the most promising (Table 2 and Figure 1A). The study conducted by Abumourad et al. (2013) in Egypt reported that *L. plantarum* CFS showed a high inhibition zone diameter (9 mm) against *Pseudomonas aeruginosa*. In our study, the CFS of L_1_ isolate presented higher antagonistic activity against an enteroaggregative *E. coli* (35 mm), as observed in Figure 1B. Additionally, it was observed that L_1_ and L_4_ recorded the largest inhibition zone diameters against all pathogens, including PDR enteroaggregative *E. coli* (35 and 37 mm, respectively) and *S. typhi* (28 mm), as shown in Table 2. Moreover, the strains L_1−5_ had also strong activity against PDR *Klebsiella pneumoniae* and *P. aeruginosa* (data not shown), indicating a broad spectrum of antibacterial activity. These results were explained by Zambou et al. (2013), who reported that bactericidal proteins with antagonistic activities were produced by some strains of lactobacilli. In the present study, in spite of neutralizing the acidic pH of the CFS and treatment with catalase enzyme, the activity was not affected significantly. On the contrary, treatment of the CFS with either trypsin or proteinase K resulted in loss of the activity (data not shown). These results suggest that the activity was not due to neither the organic acids nor the hydrogen peroxide but it might be due to the presence of bacteriocins.

**Figure 2:**
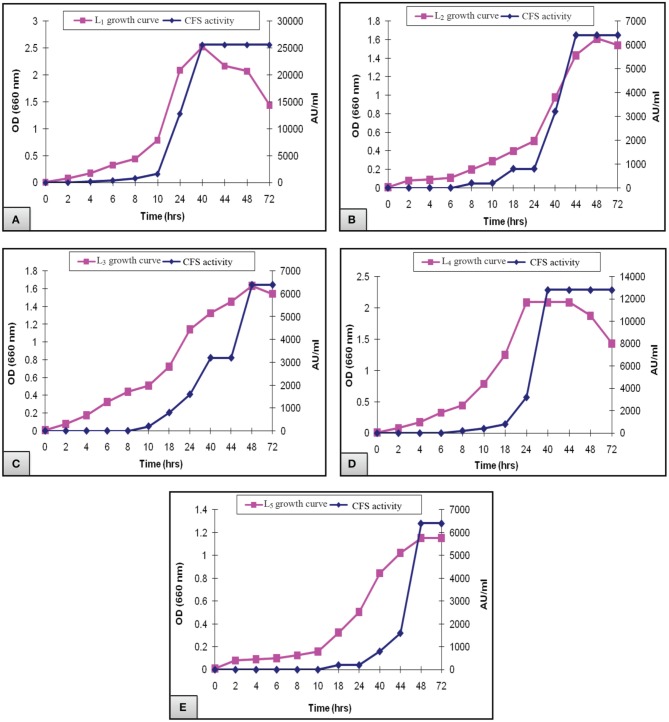
**Growth curve of *L. plantarum* (A) L_1_, (B) L_2_, (C) L_3_, (D) L_4_, and (E) L_5_ showing the biomass and CFS activity**.

Figure 2 presented the results of biomass measurements and CFS activity of the potent *L. plantarum* isolates. The range of the highest biomass (1.2–2.5) and CFS activity (6400–25,600 AU/ml) was detected at 40–48 h of incubation. Maximal CFS activity (25,600 AU/ml) was detected for L_1_ isolate at the end of the exponential growth phase and then remained constant till the end of the growth cycle (Figure 2A). The activity of L_4_ CFS (800 AU/ml) was detected starting at 10 h and then reached a maximal level (12,800 AU/ml) at 40 h (Figure 2D). The later activity remained constant up to 72 h of incubation, suggesting that extracellular proteases had not been produced. A similar result was reported by Todorov (2008). Additionally, L_2_, L_3_, and L_5_ isolates showed lower CFS activities (6400 AU/ml) at 44–48 h compared to L_1_and L_4_ isolates (Figures 2B,C,E). The study conducted by Karthikeyan and Santosh (2009), reported that the maximum activity obtained by *L. plantarum* was 3400 AU/ml in MRS broth after 14 h of incubation. Similar results were reported for bacteriocin ST151BR and plantaricin Y, ST461BZ and ST462BZ (Chin et al., 2001; Todorov and Dicks, 2004, 2005b). In addition, Todorov (2008) reported that the activity of bacteriocin AMA-K reached 25,600 AU/ml after the addition of 30 g of glucose to the medium. These findings reflect the potent antibacterial activity of our isolates, particularly the L_1_ CFS.

**Figure 3:**
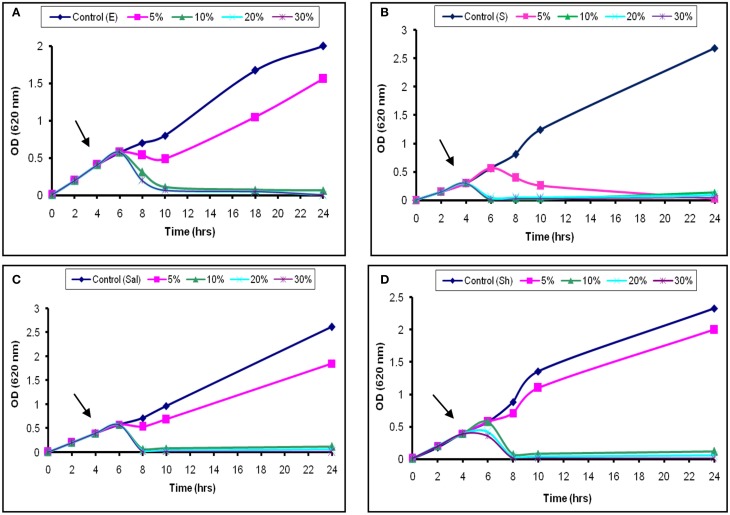
**Bactericidal activity of L_1_ CFS against indicator strains. (A)** *E. coli*, **(B)** *S. aureus*, **(C)** *S. typhi*, and **(D)** *Sh. flexneri*. The arrow indicates the point at which the CFS was added.

The inhibitory activity of the L_1_ CFS against the growth of *S. aureus* was shown in Figure 3B. Addition of 5% (52 AU/ml) was associated with a temporary increase in the OD_620_ for the next 2 h, followed by a continuous decrease. Moreover, the addition of 20% (200 AU/ml) of the CFS caused the OD_620_ to decrease until 24 h (Figure 3B). The later effect was also recorded at high concentrations used against *Sh. flexneri* (Figure 3D). Moreover, aliquots collected from the treated broth culture and inoculated on nutrient agar showed decreased viable counts compared with the control. These results suggest that the decrease in the viable count was due to bactericidal and antimicrobial rather than a bacteriostatic effect. The effects obtained with *S. typhi* and *E. coli* were similar to those found for *Sh. flexneri* (Figures 3A,C). The effect of L_2−5_ was similar to that of L_1_ (data not shown). The study conducted by Todorov (2008) reported that bacteriocin AMA-K was adsorbed at a concentration of 75% to *Listeria ivanovii* and *L. monocytogenes* cells and had a bactericidal effect. Additionally, Todorov and Dicks (2006) reported that strains sensitive to plantaricin 423 strongly adsorbed the peptide.

**Figure 4:**
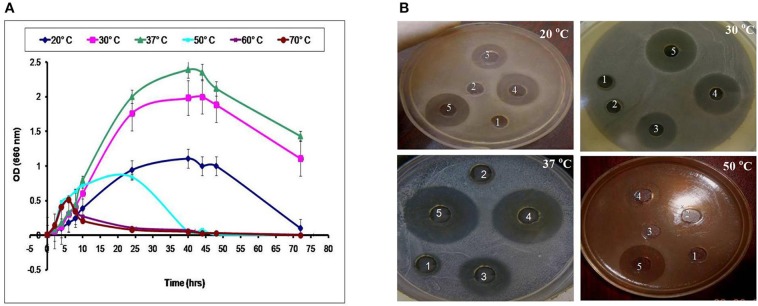
**Effect of different incubation temperatures on (A) the biomass and (B) CFS activity of the L_1_ strain against *S. aureus* at different time intervals (1, 8 h; 2, 10 h; 3, 24 h; 4, 40 h; and 5, 48 h)**.

**Figure 5:**
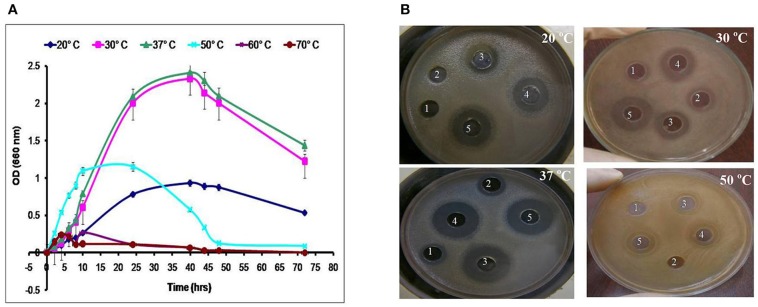
**Effect of different incubation temperatures on (A) the biomass and (B) CFS activity of the L_4_ strain against *E. coli* at different time intervals (1, 6 h; 2, 8 h; 3, 24 h; 4, 40 h; and 5, 48 h)**.

LAB have been used as probiotic microorganisms for humans. In order to reach the colon in a viable state, they must cope with specific stress challenges throughout the gastrointestinal tract including the acidic pH of the stomach and the presence of bile salts in the upper parts of the small intestine (Ruiz et al., 2013). In addition, higher temperature degrees and high salt concentrations might be faced during industrial processing. The temperature, pH and sodium chloride concentration play an important role in cell growth and bacteriocin production (Karthikeyan and Santosh, 2009). In the present study, the growth and CFS activity of L_1−5_ were investigated at different temperatures (20, 30, 37, 50, 60, and 70°C) and different time intervals. Furthermore, the maximum arbitrary unit for L_1_ was measured as 25,600 AU/ml at 37°C, and the minimum level was 200 AU/ml at 50°C (data not shown). Loss of the activity was detected at 60°C and 70°C despite the presence of biomass for at least 6 h (Figures 4, 5). Other *L. plantarum* isolates (L_2−5_) could not grow at temperatures higher than 37°C. The study conducted by Rawal et al. (2013) reported that the optimal temperature for growth and bacteriocin production was 37°C. Similarly, Karthikeyan and Santosh (2009) reported that the maximum activity of *L. plantarum* bacteriocin was 12,800 AU/ml at 40°C.

**Figure 6:**
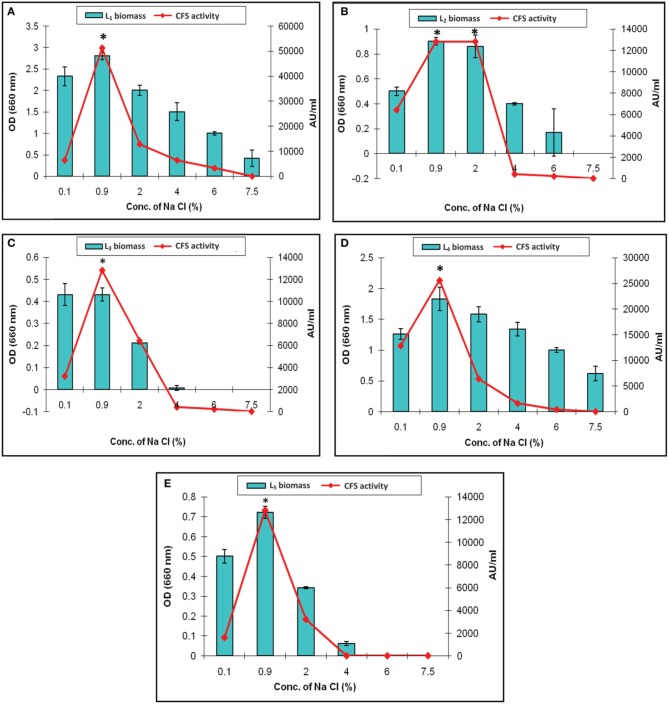
**Effect of different concentrations of sodium chloride on the biomass and CFS activity of *L. plantarum isolates*. (A)** L_1_, **(B)** L_2_, **(C)** L_3_, **(D)** L_4_, and **(E)** L_5_. ^*^Means in a given column with different superscript are significantly different (^*^p < 0.05).

The effect of various concentration of sodium chloride on the biomass and CFS activity was studied. It revealed increased arbitrary units by all of the isolates which were ranged from 12,800 to 51,200 AU/ml at 0.9% Na Cl. The maximum activity (51,200 AU/ml) was recorded by L_1_ CFS at 0.9% Na Cl. It was completely lost upon increasing the salt concentration to 7.5% as presented in Figure 6A. This finding was explained by Rawal et al. (2013), who reported that an increase in the salt concentration was associated with slow cell growth, less efficient biomass production, and subsequently low bacteriocin production because the production of bacteriocin follows primary metabolite kinetics. In our study, only the L_1_ and L_4_ isolates could resist high osmolarity (7.5% Na Cl), but no CFS activity detected (Figures 6A,D, respectively). The other isolates showed survival in the presence of 4% Na Cl with decreased CFS activity (Figures 6B,C,E). Similar results were reported by other researchers (Karthikeyan and Santosh, 2009; Rawal et al., 2013). The results indicates that the Na Cl concentration of 0.9% was optimal for CFS activity, and the only exception was L_2_ CFS, which showed maximal activity (12,800 AU/ml) in the presence of 0.9 and 2% sodium chloride (Figure 6B).

An important criterion for defining a good source of probiotics is the tolerance to the high acid levels that are present in the human stomach, for instance, pH 1.5 was the lowest value recorded during fasting. Thus, a good probiotic source must withstand low pH levels at least pH 3 as reported by Lin et al. (2006). In the present work, a general reduction in the isolates count was found after exposure to pH 1.5 and 3.0, and the count was fairly constant at pH 7.2 (control). All of the tested *L. plantarum* isolates recorded a count at 0.0 h. After 1.5 h of incubation at pH 1.5, all of the isolates showed no growth, with the exception of L_1_ and L_4_, which presented moderate counts of 6.24 ± 0.54 and 4.52 ± 0.11 log_10_ CFU/ml, respectively, and after 3 h of incubation at the same pH, the counts were markedly decreased to 5.19 ± 0.51 and 3.02 ± 0.07 log_10_ CFU/ml, respectively. The other isolates showed no growth, suggesting that they were killed by this harsh pH (Table 3). The study conducted by Mandal et al. (2006) also confirmed that the viability count of the bacteria decreased markedly after exposure to simulated gastric juice at pH 1.5 for 3 h. The analysis of pH 3 (Table 3) revealed that all of the isolates showed survival with the exception of L_2_. These results were explained by Sahadeva et al. (2011), who reported that this pH was not too low to cause complete destruction of all of the cells.

**Table 3:** **Total plate counts for the five potent *L. plantarum* isolates (L_1−5_) on MRS agars at different pH values of 1.5, 3.0, and 7.2 (control) over 1.5 h intervals**.

**pH value**	**Isolate code**	**Total plate counts (log_10_ CFU/ml)^a^**		
		**0 h**	**1.5 h**	**3.0 h**
1.5	L1	9.55 ± 0.08^b^	6.24 ± 0.54^c^	5.19 ± 0.51^d^
	L2	7.12 ± 0.05^b^	−	−
	L3	7.99 ± 0.23	−	−
	L4	9.06 ± 0.06^b^	4.52 ± 0.11^c^	3.02 ± 0.07^d^
	L5	8.45 ± 0.14	−	−
3	L1	8.25 ± 0.11^b^	8.03 ± 0.25^c^	8.01 ± 1.02^d^
	L2	7.22 ± 0.14^b^	−	−
	L3	7.75 ± 0.36^b^	2.67 ± 0.79^c^	1.27 ± 1.44^d^
	L4	9.32 ± 0.09^b^	9.08 ± 0.87^c^	9.02 ± 0.41^d^
	L5	6.17 ± 0.36^b^	5.92 ± 0.25^c^	4.56 ± 0.13^d^
7.2	L1	8.44 ± 0.28^b^	8.65 ± 047^c^	8.89 ± 0.22^d^
	L2	5.29 ± 0.77^b^	5.11 ± 0.08^c^	5.09 ± 0.74^d^
	L3	6.72 ± 0.89^b^	6.67 ± 0.02^c^	6.25 ± 0.44^d^
	L4	9.55 ± 0.03^b^	9.71 ± 0.08^c^	9.88 ± 0.05^d^
	L5	6.78 ± 0.33^b^	6.84 ± 0.77^c^	6.99 ± 0.07^d^

a Each value in the table represents the mean value ± Standard Deviation (SD). Each data point is the average of two repeated measurements from 3 independently replicated experiments, *n* = 3.

b,c,d Mean value with different superscripts in the same row differs significantly (*P* < 0.05).

^−^The hyphen symbol represents plate counts of <1 log_10_CFU/mL on the MRS agar plates.

**Figure 7:**
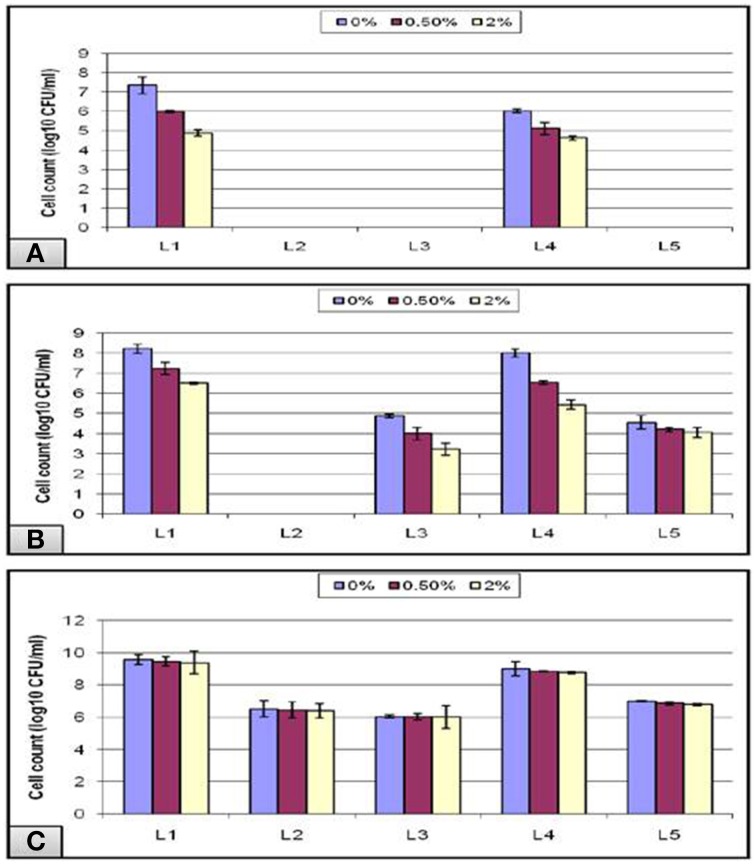
**Effect of different bile concentrations on the cell viability of *L. plantarum* (L_1−5_) isolates at (A) pH 1.5, (B) pH 3, and (C) pH 7.2 during 24 h of incubation at 37°C**.

In addition to the strong acidity of the stomach, the probiotic microorganisms taken orally have to defend against the bile salt in the gastrointestinal tract. Hence, bile tolerance is one of the most crucial properties for probiotic bacteria, as it determines its ability to survive in the small intestine, and consequently its capacity to play its functional role as a probiotic. Thus, under normal physiological conditions, our intestine holds a bile salt concentration gradient ranging from more than 40 mM to less than 1 mM—equivalent to a range between 2 and 0.05%—which is responsible, among other factors, for shaping the microbial community found in our gut (Klayraung et al., 2008; Ruiz et al., 2013). In our study, a bile concentration of 0% was used as a control for all of the experiments, and the highest growth was obtained at this concentration. The data obtained from the acid tolerance experiment showed that all of the isolates that could not survive at low pH 1.5, failed also to grow in the subsequent bile test except for L_1_ and L_4_ isolates (Figure 7A). Following pH 3, all isolates except L2 could grow in bile with a gradual decline in the viable count as the bile concentration increased (Figure 7B). In addition, all of the tubes labeled pH-7.2 showed nonsignificant gradual decrease in the viable count in spite of the increase in the bile concentration (Figure 7C).

Overall, our results showed that bile did not inhibit the bacterial growth completely even at a concentration of 2% bile as high counts of bacteria were obtained. This might be due to a stress adaptation mechanism which could explain the increased growth obtained at longer incubations following the pre-exposure to acid stress (Sahadeva et al., 2011). Another cause that may explain the stress tolerance is the presence of Tween 80, which is a nonionic surfactant and emulsifier commonly found in MRS. It has been postulated to enhance the stability of the cell membrane and thus contribute to the bile tolerance observed in some strains (Kimoto et al., 2002). In addition, Suskovic et al. (2001) reported that bile salt hydrolase (BSH) activity can account for bile salt resistance. It has been observed in some strains that BSH hydrolyzes conjugated bile, thereby reducing its bactericidal effect, and this may explain the sensitivity of some strains that lack BSH activity (Sahadeva et al., 2011).

In conclusion, the present investigation detected the potent antibacterial activity of the five *L. plantarum* isolates recovered from fermented milk samples collected in Egypt. These isolates could inhibit all tested food-borne pathogenic bacteria, and large inhibition zones were recorded compared to other studies conducted in Egypt and other countries. This work also showed that these isolates were defensive and were denoted as exceptional bacteria because of their constructive role on human pathogens. Moreover, the indicator pathogens were virulent and extensively and/or pandrug–resistant, particularly against imipenem, third- and fourth-generation cephalosporins, aminoglycosides, fluoroquinolones and vancomycin. Additionally, excellent CFS activities were recorded which might suggest the presence of bacteriocins. In addition, these strains were subjected to different stress factors, and only two strains were tolerant to the low pH in the human stomach and to bile acid. Additionally, these strains showed resistance to the high temperature (70°C) and high osmolarity (up to 7.5% Na Cl) that might be faced during industrial fermentation.

Finally, the capability of *L. plantarum* incorporated in fermented milk to inhibit the growth and even kill certain enteric and diarrhogenic pathogens suggest the health benefits that may be derived from the consumption of such products. These benefits may include protection from diarrhea, food poisoning and even systemic and enteric infections. In addition, some strains showed higher cell surface hydrophobicity that ranged from 58.1 to 73.6% when the microbial adhesion to hexadecane droplets was carried out (data not shown). This can give information about the possibility of probiotics to colonize the intestine and may modulate the host immune system. Moreover, the *L. plantarum* isolates showed absence of transmissible antibiotic resistance genes because they were susceptible to all tested antibiotics with no plasmids detected in their profiles following plasmid DNA gel electrophoresis (data not shown). Accordingly, they might be helpful in the fields of food and pharmaceutical industry because of their prophylactic and therapeutic potential.

At present, we are focusing on the purification and characterization of the substances which were responsible for the antibacterial activity so that this work is in progress.

### Conflict of interest statement

The authors declare that the research was conducted in the absence of any commercial or financial relationships that could be construed as a potential conflict of interest.
